# Supplementary material for: Unveiling the Bioactive Potential of the Invasive Jellyfish Phyllorhiza punctata Through Integrative Transcriptomic and Proteomic Analyses
Source: Biomolecules. 2025 Aug 4;15(8):1121. doi: 10.3390/biom15081121 (PMC12383608; doi:10.3390/biom15081121)
Supplement: Supplementary file 1 [file biomolecules-15-01121-s001.zip › Suplementary Material/FileS3/PEPFOLD4/XP_065063795_13099/clusters.html]

 PEP-FOLD clusters


Download archive of all models  here   

|  |  |  |
| --- | --- | --- |
| # | file | sOPEP |
| # cluster 1 - Size: 54 |  |  |
|  | XP\_065063795\_13099-model1 | -14.1583 |
|  | XP\_065063795\_13099-model1.1 | -14.1583 |
|  | XP\_065063795\_13099-model1.2 | -14.1583 |
|  | XP\_065063795\_13099-model1.3 | -14.1583 |
|  | XP\_065063795\_13099-model1.4 | -14.0847 |
|  | XP\_065063795\_13099-model1.5 | -14.0847 |
|  | XP\_065063795\_13099-model1.6 | -14.0847 |
|  | XP\_065063795\_13099-model1.7 | -14.0752 |
|  | XP\_065063795\_13099-model1.8 | -14.0752 |
|  | XP\_065063795\_13099-model1.9 | -14.0752 |
|  | XP\_065063795\_13099-model1.10 | -14.0752 |
|  | XP\_065063795\_13099-model1.11 | -14.0752 |
|  | XP\_065063795\_13099-model1.12 | -14.0752 |
|  | XP\_065063795\_13099-model1.13 | -14.0752 |
|  | XP\_065063795\_13099-model1.14 | -14.0752 |
|  | XP\_065063795\_13099-model1.15 | -14.0752 |
|  | XP\_065063795\_13099-model1.16 | -14.0752 |
|  | XP\_065063795\_13099-model1.17 | -14.0752 |
|  | XP\_065063795\_13099-model1.18 | -14.0752 |
|  | XP\_065063795\_13099-model1.19 | -14.0752 |
|  | XP\_065063795\_13099-model1.20 | -14.0752 |
|  | XP\_065063795\_13099-model1.21 | -14.0752 |
|  | XP\_065063795\_13099-model1.22 | -14.0752 |
|  | XP\_065063795\_13099-model1.23 | -14.0752 |
|  | XP\_065063795\_13099-model1.24 | -14.0752 |
|  | XP\_065063795\_13099-model1.25 | -14.0752 |
|  | XP\_065063795\_13099-model1.26 | -14.0752 |
|  | XP\_065063795\_13099-model1.27 | -14.0752 |
|  | XP\_065063795\_13099-model1.28 | -14.0752 |
|  | XP\_065063795\_13099-model1.29 | -14.0229 |
|  | XP\_065063795\_13099-model1.30 | -14.0229 |
|  | XP\_065063795\_13099-model1.31 | -14.0229 |
|  | XP\_065063795\_13099-model1.32 | -14.0229 |
|  | XP\_065063795\_13099-model1.33 | -14.0229 |
|  | XP\_065063795\_13099-model1.34 | -14.0229 |
|  | XP\_065063795\_13099-model1.35 | -14.0229 |
|  | XP\_065063795\_13099-model1.36 | -14.0007 |
|  | XP\_065063795\_13099-model1.37 | -14.0007 |
|  | XP\_065063795\_13099-model1.38 | -14.0007 |
|  | XP\_065063795\_13099-model1.39 | -14.0007 |
|  | XP\_065063795\_13099-model1.40 | -14.0007 |
|  | XP\_065063795\_13099-model1.41 | -14.0007 |
|  | XP\_065063795\_13099-model1.42 | -14.0007 |
|  | XP\_065063795\_13099-model1.43 | -13.9903 |
|  | XP\_065063795\_13099-model1.44 | -13.9903 |
|  | XP\_065063795\_13099-model1.45 | -13.9903 |
|  | XP\_065063795\_13099-model1.46 | -13.9903 |
|  | XP\_065063795\_13099-model1.47 | -13.9903 |
|  | XP\_065063795\_13099-model1.48 | -13.9903 |
|  | XP\_065063795\_13099-model1.49 | -13.9903 |
|  | XP\_065063795\_13099-model1.50 | -13.9903 |
|  | XP\_065063795\_13099-model1.51 | -13.9903 |
|  | XP\_065063795\_13099-model1.52 | -13.9903 |
|  | XP\_065063795\_13099-model1.53 | -13.9903 |
| # cluster 2 - Size: 46 |  |  |
|  | XP\_065063795\_13099-model2 | -14.0684 |
|  | XP\_065063795\_13099-model2.1 | -14.0684 |
|  | XP\_065063795\_13099-model2.2 | -14.0684 |
|  | XP\_065063795\_13099-model2.3 | -14.0684 |
|  | XP\_065063795\_13099-model2.4 | -14.0684 |
|  | XP\_065063795\_13099-model2.5 | -14.0684 |
|  | XP\_065063795\_13099-model2.6 | -14.0684 |
|  | XP\_065063795\_13099-model2.7 | -14.0684 |
|  | XP\_065063795\_13099-model2.8 | -14.0684 |
|  | XP\_065063795\_13099-model2.9 | -14.0684 |
|  | XP\_065063795\_13099-model2.10 | -14.0684 |
|  | XP\_065063795\_13099-model2.11 | -14.0684 |
|  | XP\_065063795\_13099-model2.12 | -14.0684 |
|  | XP\_065063795\_13099-model2.13 | -14.0684 |
|  | XP\_065063795\_13099-model2.14 | -14.0684 |
|  | XP\_065063795\_13099-model2.15 | -14.0684 |
|  | XP\_065063795\_13099-model2.16 | -14.0684 |
|  | XP\_065063795\_13099-model2.17 | -14.0684 |
|  | XP\_065063795\_13099-model2.18 | -14.0684 |
|  | XP\_065063795\_13099-model2.19 | -14.0684 |
|  | XP\_065063795\_13099-model2.20 | -14.0684 |
|  | XP\_065063795\_13099-model2.21 | -14.0684 |
|  | XP\_065063795\_13099-model2.22 | -14.0226 |
|  | XP\_065063795\_13099-model2.23 | -13.9692 |
|  | XP\_065063795\_13099-model2.24 | -13.9659 |
|  | XP\_065063795\_13099-model2.25 | -13.9659 |
|  | XP\_065063795\_13099-model2.26 | -13.9659 |
|  | XP\_065063795\_13099-model2.27 | -13.9659 |
|  | XP\_065063795\_13099-model2.28 | -13.9659 |
|  | XP\_065063795\_13099-model2.29 | -13.9659 |
|  | XP\_065063795\_13099-model2.30 | -13.9659 |
|  | XP\_065063795\_13099-model2.31 | -13.9659 |
|  | XP\_065063795\_13099-model2.32 | -13.9659 |
|  | XP\_065063795\_13099-model2.33 | -13.9659 |
|  | XP\_065063795\_13099-model2.34 | -13.9659 |
|  | XP\_065063795\_13099-model2.35 | -13.9659 |
|  | XP\_065063795\_13099-model2.36 | -13.9378 |
|  | XP\_065063795\_13099-model2.37 | -13.9378 |
|  | XP\_065063795\_13099-model2.38 | -13.9378 |
|  | XP\_065063795\_13099-model2.39 | -13.9378 |
|  | XP\_065063795\_13099-model2.40 | -13.9378 |
|  | XP\_065063795\_13099-model2.41 | -13.9378 |
|  | XP\_065063795\_13099-model2.42 | -13.9378 |
|  | XP\_065063795\_13099-model2.43 | -13.9378 |
|  | XP\_065063795\_13099-model2.44 | -13.9378 |
|  | XP\_065063795\_13099-model2.45 | -13.9221 |
|  |  |  |
